# Biomarkers of PTSD: military applications and considerations

**DOI:** 10.3402/ejpt.v5.23797

**Published:** 2014-08-14

**Authors:** Amy Lehrner, Rachel Yehuda

**Affiliations:** 1 James J. Peters Veterans Affairs Medical Center, Bronx, NY, USA; 2 Departments of Psychiatry and Neuroscience, Icahn School of Medicine at Mount Sinai, New York, NY, USA

**Keywords:** Posttraumatic stress disorder, biomarkers, clinical utility, ethics, translation

## Abstract

**Background:**

Although there are no established biomarkers for posttraumatic stress disorder (PTSD) as yet, biological investigations of PTSD have made progress identifying the pathophysiology of PTSD. Given the biological and clinical complexity of PTSD, it is increasingly unlikely that a single biomarker of disease will be identified. Rather, investigations will more likely identify different biomarkers that indicate the presence of clinically significant PTSD symptoms, associate with risk for PTSD following trauma exposure, and predict or identify recovery. While there has been much interest in PTSD biomarkers, there has been less discussion of their potential clinical applications, and of the social, legal, and ethical implications of such biomarkers.

**Objective:**

This article will discuss possible applications of PTSD biomarkers, including the social, legal, and ethical implications of such biomarkers, with an emphasis on military applications.

**Method:**

Literature on applications of PTSD biomarkers and on potential ethical and legal implications will be reviewed.

**Results:**

Biologically informed research findings hold promise for prevention, assessment, treatment planning, and the development of prophylactic and treatment interventions. As with any biological indicator of disorder, there are potentially positive and negative clinical, social, legal, and ethical consequences of using such biomarkers.

**Conclusions:**

Potential clinical applications of PTSD biomarkers hold promise for clinicians, patients, and employers. The search for biomarkers of PTSD should occur in tandem with an interdisciplinary discussion regarding the potential implications of applying biological findings in clinical and employment settings.

There has been extensive progress in characterizing the biological basis of posttraumatic stress disorder (PTSD). Perturbations in the hypothalamic–pituitary–adrenal (HPA) axis, sympathetic adrenomedullary system, and alterations in brain structure and function have been associated with risk for development of PTSD following trauma exposure, with PTSD symptoms and diagnosis, and with recovery (e.g., Pitman et al., 2012; Schmidt, Kaltwasser, & Wotjak, 2013). In a new era of “big” data that allows for examination and detection of genome-wide genomics, transcriptomics, proteomics, and metabolomics in conjunction with brain imaging data, there is seemingly endless opportunity for new discovery. As the field moves closer to identifying biomarkers based on established criteria, it is important to anticipate how such markers would be used (Yehuda, Neylan, Flory, & McFarlane, 2013). These questions are particularly relevant for the military (prevention) and the Veteran's Administration (treatment), which represent institutions in a position to take leadership in this arena.

Although many biological alterations have been observed in association with PTSD, no current biological variable has yet passed the threshold for a reliable and specific PTSD biomarker (for recent reviews of potential biomarkers of PTSD, see Baker, Nievergelt, & O'Connor, 2012; Bomyea, Risbrough, & Lang, 2012; DiGangi et al., 2013; Schmidt et al., 2013; Zoladz & Diamond, 2013). A disease biomarker refers to “a characteristic that is objectively measured and evaluated as an indicator of normal biological processes, pathogenic processes, or pharmacological responses to a therapeutic intervention” (Biomarkers Definitions Working Group, 2001). It may be that both etiologically and phenotypically PTSD does not represent a single construct, and that this complexity has confounded efforts to identify robust biomarkers. However, the potential of advanced computational and multisystemic approaches to identify the influence of multiple relevant networks may lead to important breakthroughs. Increasingly, a systems biology approach that takes advantage of innovations in integrative and computational biology is being pursued in order to characterize the complex interplay of multiple biological levels (molecular, cellular, etc.) with environmental and psychological stimuli. Extant biological research on PTSD suggests that information obtained from any given system or dimension of data alone may yield a biomarker relating to some aspect of PTSD, but that a comprehensive understanding of this condition may only be obtained from evaluating entire biological networks that in turn increase or decrease the risk of illness or affect illness severity. Thus, PTSD symptoms may be best conceptualized as emergent properties of complex networks, as opposed to core biological processes associated with a disease driven by a small number of genes. Such a multifaceted and dynamic conceptualization of PTSD risk and illness networks or pathways may require a shift away from a more narrowly defined “biomarker” of PTSD, and imply the need for more nuanced interpretations and clinical applications of identified PTSD networks. Regardless, the identification of biological systems associated with risk and disorder holds promise for the development of diagnostic tests, prognostic indicators, and prophylactic and treatment interventions, and such biological research in PTSD has been supported by stakeholders such as the U.S. Departments of Defense and Veteran's Affairs with an eye toward translational applications.

Biological measures of PTSD should reflect predictive markers of risk/resilience (pre- or posttrauma exposure), or disease markers indicating diagnostic status or symptom severity. More refined applications include prognostic markers of therapy response that may inform treatment choice or monitor response (Schmidt et al., 2013). Once the biological correlates of these constructs are identified, such biomarkers may help identify those at highest risk following trauma exposure, target prevention efforts, aid in diagnosis, treatment planning, and recovery assessment for patients, and ultimately inform the development of safe and effective pharmacological treatments for PTSD. This article will review the possible applications and implications of PTSD biomarkers, with an emphasis on military applications (see Fig. 1 for a schematic overview). Despite the significant interest in the identification of biomarkers of PTSD, there has been relatively little discussion in the literature regarding the potential uses of such biomarkers or the social, ethical, or legal implications of psychiatric biomarkers in general, and PTSD in particular (Lakhan, Vieira, & Hamlat, 2010; Singh & Rose, 2009).

**Fig. 1:**
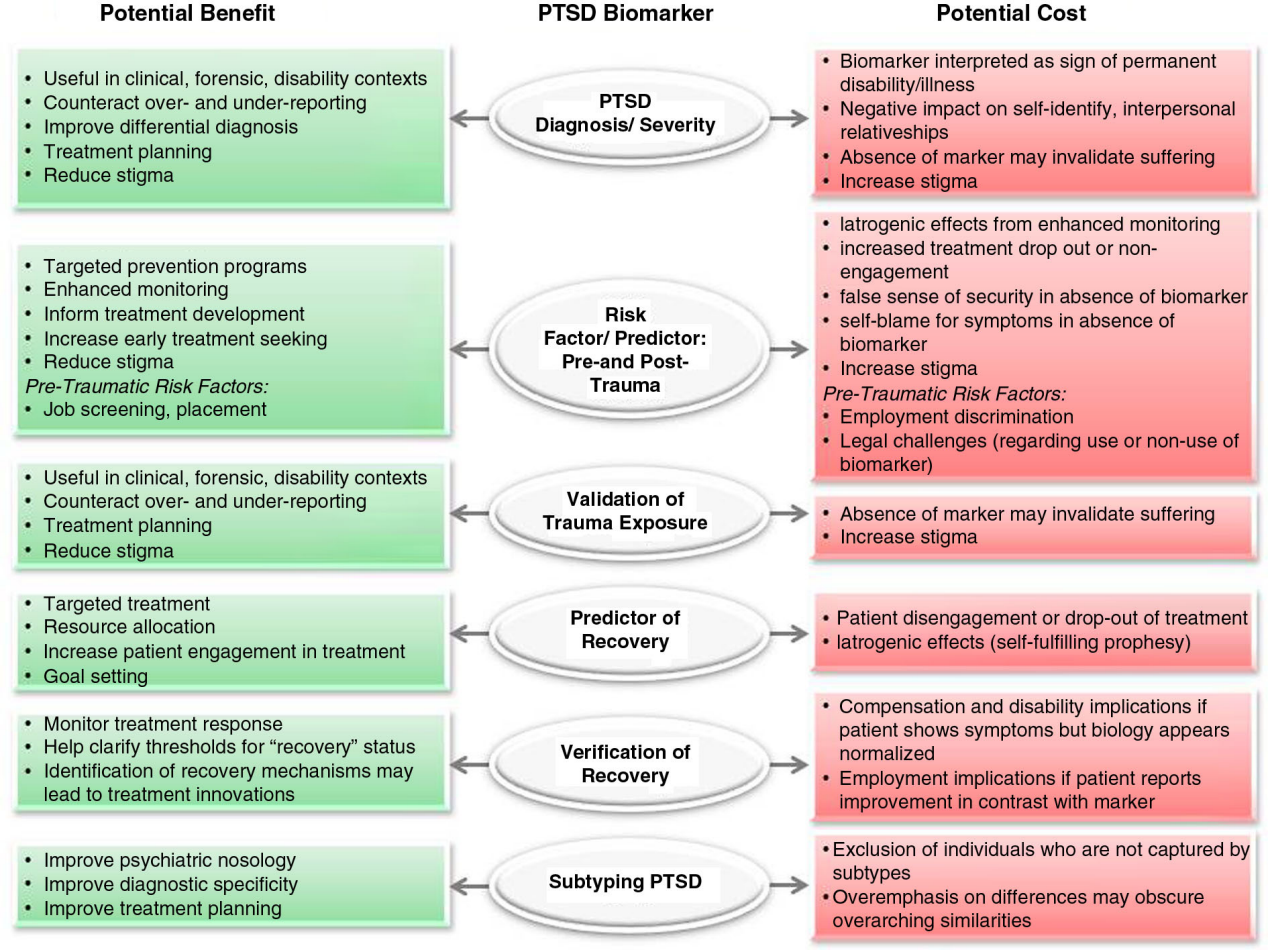
Schematic representation of potential applications of PTSD biomarkers.

## Biomarkers of PTSD symptoms or diagnosis

A biomarker that objectively confirms PTSD diagnosis and/or symptom severity should be applicable in clinical, forensic, and disability/compensation contexts or in any setting where there may be a need to verify symptoms. These include contexts where patients may deny, minimize, or exaggerate symptoms, have difficulty describing symptoms, or have difficulty attributing symptoms to trauma exposure. For example, there may be significant consequences of a positive PTSD diagnosis for disability and compensation entitlements, which may influence overreporting of symptoms, whether consciously or not. In other cases, service members may deny or minimize symptoms out of concerns over duty assignments and promotion both during military service and following discharge for veterans seeking employment in law enforcement, security, or other occupations involved in public safety and crisis response. Admitting the presence of PTSD symptoms such as extreme irritability and angry outbursts or suicidality could affect employment in work that requires carrying a weapon, for example. The presence of PTSD may also be used as a mitigating influence in court cases involving violent or aggressive behavior, or substance abuse–related problems (such as driving while drunk), both of which are common among service members with PTSD. Indeed, PTSD is one of the most litigated mental disorders in civilian settings (Bottalico & Bruni, 2012).

Accurate diagnosis is also essential for treatment planning. A biomarker of PTSD may be useful as an adjunct to clinical interview, just as lab tests are informative in medicine. While most clinicians can accurately diagnose PTSD in patients with clear trauma exposure and willingness to report symptoms, differential diagnosis with PTSD can be challenging, especially in the context of comorbid diagnoses, mild traumatic brain injury (TBI), potentially overlapping symptoms (e.g., irritability, anhedonia, sleep problems, problems with concentration, suicidality, interpersonal and relationship problems), and complicated lifecourse trajectories and stressors. The degree of symptom overlap and comorbidity between PTSD and other common sequelae of trauma such as major depressive disorder (MDD) poses challenges to reliable diagnosis. Epidemiological studies consistently show that 75–88% of adults and adolescents with PTSD meet criteria for at least one other psychiatric disorder, most often major depression (Brady, Killeen, Brewerton, & Lucerini, 2000; Breslau, Davis, Andreski, & Peterson, 1991; Creamer, Burgess, & McFarlane, 2001; Kessler, Sonnega, Bromet, Hughes, & Nelson, 1995; Kilpatrick et al., 2003). There is now an unprecedented number of veterans of the wars in Iraq and Afghanistan who present for diagnosis and treatment with histories of co-occurring trauma and blast injury exposures, estimated at 12–23% (Hoge et al., 2008; Schneiderman, Braver, & Kang, 2008; Terrio et al., 2009).

An objective lab test for PTSD could also reduce the stigma associated with a PTSD diagnosis, which creates a barrier to help-seeking and causes additional suffering for patients and families. This may be especially relevant in cultures in which any mental health problems could be perceived as signs of weakness or shame, such as military and ethnic minority communities. For example, among military personnel and veterans, the absence of objective validation of PTSD as a legitimate war-related injury can fuel the perception that PTSD and other deployment-related mental injuries are not “real.” The fact that not all warfighters develop PTSD can lead to misperceptions that such symptoms are not about the battlefield, but reflect constitutional weakness best overcome with mental discipline rather than mental health treatment. This stigma can create a divide between soldiers and the social support networks crucial to their recovery. Alternatively, some may interpret the presence of a biomarker of PTSD to mean that a potentially temporary injury will never heal.

## Biomarkers as predictors or risk factors for PTSD

Some biomarkers may be indicative of current psychopathology (i.e., a state marker), whereas others may reflect pre-traumatic risk. It is also useful to distinguish pre-traumatic from posttraumatic markers of risk. Both types have potential applications for secondary prevention efforts. Secondary, or “indicated,” prevention is a public health approach that focuses specifically on a population known to be at risk of developing a condition or disorder. Identification of pre-trauma risk markers (e.g., prior trauma exposure, personality traits, gene expression patterns, neurological structure) might lead to the development of programs that minimize the chance of exposure, or that focus early intervention resources on higher risk individuals following trauma exposure, whereas post-exposure risk markers (e.g., dynamic changes reflecting neurological adaptation after trauma) would suggest immediate intervention or monitoring (i.e., “watchful waiting”).

A biomarker that elucidates some of the pathways through which risk is conferred might also inform the development of prevention efforts targeting those pathways through environmental or pharmacologic interventions. For example, low cortisol levels in the immediate aftermath of trauma have been found to predict the development of PTSD (Delahanty, Nugent, Christopher, & Walsh, 2005; Delahanty, Raimonde, & Spoonster, 2000; Yehuda, McFarlane, & Shalev, 1998) as have pre-exposure dysregulations along the glucocorticoid signaling pathway (Van Zuiden, Kavelaars, Geuze, Olff, & Heijnen, 2013). Should the literature support such findings, pharmacologic interventions that regulate cortisol or influence the HPA axis may ultimately be developed as prophylactic measures (Delahanty et al., 2013; Yehuda & Golier, 2009). Indeed, Zohar et al. (2011) report a reduced risk for the development of PTSD among patients who received a single, high dose of hydrocortisone within 6 hours of a traumatic event. Cognitive and/or behavioral interventions might also be developed that re-regulate disordered neuroendocrine markers, or that function to counteract the risk conferred by the dysregulation of the HPA axis. Cognitive and/or behavioral correlates of such neuroendocrine risk factors might also be identified and targeted.

## Biomarkers to validate exposure

In certain circumstances, it may be useful to have access to biomarkers related to trauma exposure rather than to the development of PTSD. Certainly, the majority of patients are able to accurately report their history of trauma exposure for purposes of diagnosis, case formulation and treatment planning. However, in cases when it is difficult to establish trauma exposure, or when the exposure itself is contested, a biologic marker of trauma exposure may have clinical utility. For example, children exposed to trauma, particularly abuse at the hands of a parent or guardian, may be unable or unwilling to disclose that information. Of course, a neurobiological marker that identified trauma exposure would not replace the need for clinical sensitivity regarding the undisclosed issue, but an awareness of the potential role of trauma in the etiology of the presenting problem may nonetheless be helpful for a clinician. Individuals with a stake in documenting their trauma exposure (e.g., a rape victim, a battered woman involved in a contentious legal case) could also potentially benefit from an objective marker of traumatization.

Although this is conceptually a straightforward category, an important feature of the stress response system is that most biological changes resulting from psychological trauma exposure are transient. Thus, unless there are clear demonstrations of chronic and enduring manifestations, biomarkers of exposure may not be possible. A large meta-analysis of cross-sectional data found that trauma-exposed individuals demonstrated enhanced negative HPA axis feedback comparable to those with PTSD (Morris, Compas, & Garber, 2012). However, trauma-exposed persons had morning cortisol levels and daily cortisol output similar to trauma-unexposed controls. Hair cortisol has also been investigated as a potential biomarker of trauma exposure. For example, trauma-exposed individuals with and without PTSD had lower hair cortisol concentrations than healthy, unexposed individuals (Steudte et al., 2013). Recent research in epigenetics has shown enduring molecular changes in response to early life adversity, but a biomarker reflecting such changes would still be difficult to definitively link with a specific event. Furthermore, a biomarker of exposure should be able to distinguish the effects of trauma exposure from the long-term alterations associated with chronic or severe stress (Miller, 2008).

## Predictors of recovery and verification of recovery/remission

Neurobiological studies of PTSD might identify biomarkers that predict treatment response, a clinical application of significant utility. Such biomarkers might be used to predict response to treatment, or to different types of treatment (i.e., a moderator), as well as to monitor treatment response. Given the time- and resource-intensive nature of exposure therapies for PTSD, and since not all patients will recover from these treatments, the ability to predict which patients are likely to respond to any (or specific) treatments could facilitate treatment planning and optimize resource allocation. For example, preliminary data show that cytosine methylation and expression of the GR gene at pre-treatment predicted PTSD status following Prolonged Exposure therapy (Yehuda, Daskalakis, et al., 2013). Imaging studies have also shown that larger anterior cingulate cortex volume, decreased right amygdala activity, and increased anterior cortex activity associate with better response to cognitive behavioral therapy, whereas LL 5HTTLPR genotype, BDNF serum levels, and differential conditioned fear response associate with better SSRI/SNRI response (Schmidt et al., 2013). Such stratification markers might lead to improved clinical and economic outcomes. Biological measures should be added to treatment trials to further examine predictors of treatment response and elucidate underlying mechanisms of pathophysiology.

Biomarkers of treatment response (or risk) may also have utility not only in guiding the clinician's treatment choice, but they may also influence patient behavior (Perlis, 2011). Patients known to be at risk for PTSD may be more likely to recognize early signs of disorder and to seek help. Predictive and prognostic indicators may also increase treatment adherence, enhancing motivation to remain in even difficult therapies, such as those involving exposure to painful memories or anxiety-producing situations. Prognostic indicators may also help patients set realistic goals and to plan for the future. The risk of such markers is that indicators of poor prognosis or treatment response may contribute to increased drop-out and decreased treatment adherence, becoming self-fulfilling prophesies.

Consensus regarding the definition of recovery (or meaningful improvement) and assessment of recovery status is important for rigorous evaluations of PTSD therapies and for clinical decision-making regarding ongoing treatment planning. However, the treatment outcome literature has yet to coalesce around a universally accepted, objective measure of recovery from PTSD. As a result, “recovery” is often defined differently across studies. In its review of the PTSD treatment literature, the Institute of Medicine (IOM) Committee on Treatment of Posttraumatic Stress Disorder (2008) identified inconsistencies in defining and assessing recovery as a critical problem in the literature. Further complicating assessment of recovery are findings that patient self-report and clinician ratings may not necessarily correlate, or that there can be recovery in one domain but not another. A biomarker of recovery could significantly inform the determination of recovery status. For example, neurobiological research has identified psychotherapy-related changes in the hippocampus and hypothalamus at posttreatment (Barsaglini, Sartori, Benetti, Pettersson-Yeo, & Mechelli, 2014).

A recovery biomarker might also contribute to our understanding of the underlying mechanisms of the disease and recovery processes (which themselves might be targeted for intervention). A recovery biomarker may associate with state (i.e., may be altered at baseline as well), or it may emerge posttreatment. In the first case, it reflects a normalization of biological systems that were altered in association with PTSD, in the second it may reflect the recruitment of other systems or brain regions to compensate for PTSD-related impairments (Mechelli, 2010). A number of studies have used neuroendocrine measures of treatment response and provided some proof of concept. For example, Olff, De Vries, Guzelcan, Assies, and Gersons (2007) found an increase in dehydroepiandrosterone (DHEA) levels among those who responded to treatment for PTSD. Another study found that non-responders to treatment for PTSD resulting from the 9/11 World Trade Center terrorist attacks had significant declines in posttreatment cortisol levels that distinguished them from responders (Yehuda et al., 2009). A case study of a female PTSD patient reported an increase in basal cortisol levels and more attenuated cortisol suppression on the dexamethasone suppression test in association with symptom reduction following EMDR treatment (Heber, Kellner, & Yehuda, 2002). Recently, a pilot study of combat veterans treated with Prolonged Exposure therapy found decreased methylation of the FKBP5 gene (FKBP51) exon 1 promoter region in association with recovery (Yehuda, Daskalakis, et al., 2013).

## Subtyping of PTSD

Biomarkers of PTSD have the potential to inform debates regarding subtypes of PTSD, potentially identifying distinct biological signatures with implications for diagnosis, treatment planning and development of new interventions. For example, the construct of complex PTSD (also called Disorders of Extreme Stress Not Otherwise Specified, or DESNOS), has been researched and reported in the literature, but is not codified in the DSM or the ICD, and remains a subject of debate (e.g., Cloitre, Garvert, Brewin, Bryant, & Maercker, 2013; Ford, 1999; Friedman, Resick, Bryant, & Brewin, 2011; Van der Kolk, Roth, Pelcovitz, Sunday, & Spinazzola, 2005). Proponents argue that complex PTSD results from prolonged and repeated interpersonal trauma, usually experienced in childhood, characterized by a loss of control and absence of a means of escape (e.g., Herman, 1992). These forms of trauma may induce sequelae that are distinct from those resulting from single-episode or more circumscribed trauma (e.g., car accident or rape), such as a more diffuse and complex symptom presentation, characterological disturbances in interpersonal relatedness and self-concept, dissociation, affective dysregulation, and patterns of self-harm including self-injurious behavior and vulnerability to repeated abuse (Cloitre et al., 2013; Pelcovitz et al., 1997). Those who argue for the validity of the construct argue that patients with complex PTSD require specialized trauma-focused treatment, and that conventional treatments for PTSD may have poorer outcomes or at worst have iatrogenic effects for these patients (Ford & Kidd, 1998; Van der Kolk, 2002).

In addition to complex PTSD, personality-based PTSD subtypes along the internalizing/externalizing dimension have been proposed based on latent class analyses (Dalenberg, Glaser, & Alhassoon, 2012). Those in the internalizing class have higher levels of comorbid anxiety and depression, whereas those in the externalizing class have more significant problems with anger, aggression, and substance abuse (Forbes, Elhai, Miller, & Creamer, 2010; Wolf et al., 2012). While biological studies of these subtypes have been limited, analyses of their genetic structures using twin data from Vietnam-era veterans have found different genetic risk factors for the internalizing/externalizing dimensions, as well as a common genetic factor that increases risk for comorbidity across dimensions (Wolf et al., 2010).

Clinicians have also observed that certain patients with PTSD present primarily with anxiety and fear responses (consistent with theories of the development, maintenance, and treatment of PTSD), while others present with primary emotions such as anger, guilt, or shame. These clinical presentations may simply reflect different phenotypes of a single, underlying PTSD construct, or they may be better understood as subtypes or as distinct clinical phenomena. Finally, the research literature is inconclusive regarding the degree to which PTSD is the same phenomenon across different populations, such as civilian rape survivors and combat veterans (IOM, 2008). It is unknown whether heterogeneity in the nature and chronicity of the traumatic stressors and in potentially relevant population characteristics such as age, race, ethnicity, sex, education, and veteran cohort leads to different PTSD subgroups.

Highlighting the relevance of biological markers to inform psychiatric nosology, such research supported the reclassification of PTSD in DSM-V (American Psychiatric Association, 2013) from the anxiety disorders to a new classification, “Trauma- and Stressor-Related Disorders.” Biological findings indicating that the dissociative subtype of PTSD may represent different biological processes (Lanius et al., 2010; Mickleborough et al., 2011) also informed the addition of a dissociative specifier to the DSM-V PTSD diagnosis.

## Challenges in identifying biomarkers of PTSD

There are many challenges to the identification of valid, reliable PTSD biomarkers. Although many biological abnormalities have been associated with PTSD, including HPA axis and neurobiological alterations, there is significant heterogeneity in the literature (e.g., Zoladz & Diamond, 2013). PTSD is a multifaceted psychological disorder with symptoms across four clusters and multiple longitudinal trajectories (Bonanno et al., 2012), encompassing heterogeneous phenotypes. Furthermore, biological (genetic, epigenetic, endocrine, immunological, neurological), environmental (nature and developmental timing of trauma), psychological (cognitive, emotional, behavioral), and social vulnerability factors likely interact in complex ways to increase risk for PTSD. Given the diagnostic heterogeneity of PTSD and its multifactorial etiology, it is unlikely that a single biomarker will be identified (Schmidt et al., 2013; Zoladz & Diamond, 2013).

Furthermore, even if a marker or panel of markers was reliably observed at a given level in people with PTSD, an additional issue is whether the marker is unique to PTSD (versus a marker of trauma exposure, TBI, or other psychiatric disorder). Other challenges include whether and how comorbidities such as depression and TBI, the degree of symptom severity, or the degree of symptom improvement over time affect the biological signal. Finally, much research on PTSD uses civilian samples who have experienced a single traumatic event, such as an accident or assault. For example, of the 11 PTSD studies identified that incorporate brain imaging pre-and post-psychotherapy, only one uses a military sample (Aupperle et al., 2013; Bryant, Felmingham, Kemp, et al., 2008; Bryant, Felmingham, Whitford, et al., 2008; Cisler et al., 2014; Falconer, Allen, Felmingham, Williams, & Bryant, 2013; Farrow et al., 2005; Felmingham et al., 2007; Lindauer et al., 2008; Peres et al., 2007, 2011; Roy et al., 2010). This population may differ from the military population both in terms of baseline demographic and psychological characteristics (e.g., age, gender, personality) and in the types and chronicity of trauma they have experienced. Biomarkers for use in the military must be validated in military samples.

With the exception of strongly genetically determined medical or psychiatric disorders (e.g., Huntington's disease), biomarkers of disease reflect relative risk for disorder (or recovery). The probabilistic nature of such markers raises the question of the appropriate threshold for establishing a valid and reliable biomarker. While there are established frameworks for identifying and validating novel biomarkers, the process of translation to clinical applications requires consideration of accuracy, positive and negative predictive values, and the receiver operating characteristic (ROC) curve for any test (Fuzery, Levin, Chan, & Chan, 2013; Perlis, 2011). As noted by Perlis (2011), the criteria for the validity and utility of a test depend on how it will be used, so that determining thresholds for clinical utility *a priori* is ill-advised. The potential utility of a putative biomarker depends on many considerations, including the consequence of a false positive or false negative on a test, or the potential of the biomarker to improve prediction or classification in conjunction with existing clinical indicators (Perlis, 2011). For example, a highly accurate test may be informative in a clinical context but inadequate in a legal one.

## Clinical, ethical, social, and legal considerations in the use of PTSD biomarkers

Whereas the above discussion highlights the numerous areas in which a PTSD biomarker might have therapeutic, social, and practical benefits for veterans, clinicians, and institutions such as the military, it is incumbent on the field to consider potentially detrimental outcomes as well. Just as the identification of PTSD biomarkers may reduce stigma and legitimize suffering for some, biomarkers of disorder may increase stigma for others. The knowledge that there is a biological basis to one's expressed PTSD, or that one is at high risk for disorder following trauma exposure, may negatively affect self-identity and influence social relationships by influencing others’ perceptions (Singh & Rose, 2009). Military service members who already feel unalterably damaged by their traumatic experiences may register the presence of a biological indicator of PTSD as a physical verification that they are “damaged goods” and fundamentally different from others. Such an interpretation could have the unintended consequence of creating hopelessness and disinvestment in therapy for those who believe they are permanently marked and cannot be healed. Conversely, for those experiencing distressing and disabling symptoms, a “negative” result may invalidate their suffering and potentially affect compensation claims. For warfighters steeped in a military culture that values toughness, discipline, and willpower, the absence of objective verification of disorder may lead to rejection or judgment by superior officers, soldiers, and even family.

Singh and Rose (2009) caution against the risk of reductionist and oversimplistic understandings and applications of psychiatric illness biomarkers. It will be easy for employers and lay people to overvalue an objective biological marker, and the use of a test for a biomarker of disorder must be accompanied by information and education to all users of the test and to the public regarding the probabilistic nature of such indicators. As with any medical test, a test for a PTSD biomarker will always have the risk of false positives and false negatives. One can have prostate cancer with a normal PSA level, for example. Conversely, the presence of the BRCA1 gene does not signify the presence of breast cancer. As with other forms of psychological assessment or testing, ethical imperatives of testing for PTSD biomarkers include feedback about the nature of the test results and discussion of the idiosyncratic meaning for the individual. Furthermore, it will be important not to oversimplify the disorder itself and equate it with a single biological alteration. The principle of equifinality suggests that there may be diverse biological networks or systems implicated in PTSD. Indeed, brain imaging research led to the recognition of a dissociative subtype of PTSD with different phenotypic and biological characteristics, as noted above (Lanius et al., 2010).

With regard to biological markers of risk, the potential misunderstanding and misapplication of such markers is even greater given the probabilistic nature of such indicators. It is unclear how screening for biomarkers of risk will affect employment where risk of trauma exposure is high, such as military, law enforcement, or emergency response contexts. How much should the presence of risk factors influence decisions about who should serve and in what capacity, or whether those who experience a critical incident should be returned to the battlefield or to work? Since trauma exposure is a precondition for PTSD, and since the costs of PTSD are high (both for the individual and the organization), will employers choose to simply screen out all candidates with risk markers, or will such indicators inform job placement or lead to assignments that reduce risk for exposure (such as non-combat or desk-based jobs)? Using biomarkers to screen applications may protect the institution from long-term financial costs associated with compensation claims, poor performance, and potential legal liability. Such use may also protect soldiers, their families, and the community from the psychological and economic costs of PTSD, as well as the consequences for public safety. On the other hand, such screening restricts individual rights to employment and service based on outcomes that may not occur. Furthermore, mandatory screening raises questions of consent and confidentiality (Caux, Roy, Guilbert, & Viau, 2007).

Such applications could lead to claims of discrimination in hiring or job placement. Concerns regarding potential discrimination, particularly in employment and insurance, have led to legislative and policy efforts to prohibit or limit such use of biological data. In the United States, the Genetic Nondiscrimination Act of 2008 prohibits discrimination based on “genetic information” (including RNA or chromosomal changes), but does not encompass any other potential biomarkers. Several European countries have also enacted specific legislation prohibiting genetic discrimination in specific circumstances, including Belgium, Austria, Denmark, France, Germany, Lithuania, Norway, Portugal, and Sweden (Otlowski, Taylor, & Bombard, 2012). There are two bills currently pending in Canada to amend the Canadian Human Rights Act to prohibit discrimination based on genetic data. International efforts to address risks for discrimination include the United Nations Educational, Scientific, and Cultural Organization (UNESCO) Universal Declaration on the Human Genome and Human Rights (1997) and International Declaration on Human Data (2003), which prohibit genetic discrimination. The Council of Europe's influential European Convention on Human Rights and Biomedicine (1997), known as the Oviedo Convention), prohibits discrimination based on genetic heritage, and the European Union's Charter of Fundamental Rights (2000) includes genetic data in its nondiscrimination clause. Other efforts to reduce risks of genetic discrimination include the implementation of guidelines and code of practice, voluntary moratoria, and the development of independent bodies with monitoring or policy advocacy agendas (Otlowski et al., 2012). These efforts are all specific to discrimination based on genetic data, but the potential for discrimination based on non-genomic biomarkers for PTSD (or other medical illnesses or conditions) remains to be addressed.

Should any qualified applicant have the right to serve, or do risk factors for PTSD constitute a legitimate cause for rejection? It is not clear whether such biomarkers would have a similar status to personality characteristics, for example, that associate with increased risk or poor performance and which are currently deemed relevant for employment in high-risk jobs, such as law enforcement. On the other hand, the identification of pre-exposure risk markers may also raise legal liability issues for an employer who places a high-risk employee in an environment where trauma exposure is likely. For example, British combat veterans brought one of the largest personal injury suits in UK history against the United Kingdom's Ministry of Defense for negligence in failing to “screen out vulnerable individuals” and to “debrief them properly,” among other things (Dyer, 2002).The identification of risk factors may be used proactively to target service members for resilience training pre-deployment, or for “watchful waiting” following trauma exposure, but the potentially negative consequences of identifying service members with a PTSD risk factor must be considered. How might the identification of some as higher risk for PTSD affect unit cohesion and trust among soldiers? The potential for stigma, with its myriad consequences, is high. Enhanced monitoring of those at risk following trauma exposure also raises the specter of iatrogenic effects. For those who develop PTSD and are found to have pre-existing biological risk factors, might this be used to deny or minimize claims for compensation and benefits? A biomarker of pre-traumatic risk might be taken as a sign of weakness that invalidates the profound impact of combat and the suffering and impairment associated with PTSD. Baum and Savulescu (2013) argue that once biomarkers of risk have been identified, the moral landscape changes, and the ethical burden shifts so that *not* using biological information requires justification. How to use that information, however, remains to be established.

Furthermore, it is increasingly clear that “risk” is not a static category in PTSD, when risk for disorder changes with multiple deployments or exposures, for example (Yehuda, Neylan, et al., 2013). Markers of “resilience” also do not necessarily permanently protect against illness. This means that tests for risk might need to be administered periodically to those chronically exposed to traumatic stressors to account for “wear and tear.” Individuals who show biomarkers of resilience, or who lack the risk biomarkers, may experience a false sense of security or blame themselves should they become symptomatic (Silver & Sharp, 2006). The identification of dynamic biological networks that are sensitive to ongoing perturbations in the environment may elucidate how biology changes over time and in response to life events to increase (or decrease) risk of symptoms. To use a risk marker as a screening tool may overestimate and overvalue its influence and discount the potential for individual resilience. It may be that risk markers are most valuable in the study of PTSD pathophysiology in support of development of new preventative and treatment interventions.

The use of biomarkers of recovery and treatment response also raises social, ethical and legal issues. What might the implications be for compensation and benefits if an individual shows normalization in biological indicators of PTSD following treatment but continues to report distressing or impairing symptoms? For example, a recent fMRI study of psychotherapy for PTSD in veterans found improvement in cerebral function following treatment while PTSD symptom reduction as assessed by the Clinician Administered PTSD Scale for DSM-IV (CAPS) was modest (Roy et al., 2010). Conversely, how might it affect a service member's job posting, chance of promotion, or sense of self if he or she reports improvement but continues to evidence biomarkers of disorder? Prognostic indicators may help guide treatment choice, but might they also become a self-fulfilling prophesy for those with biomarkers of treatment resistance? As the pursuit of PTSD biomarkers continues, a parallel conversation regarding ethical and legal implications is needed.

## Conclusions

It is tempting to think that the application of biological information to clinical and employment-related decision-making will be a straightforward good, and there are clearly many potentially beneficial applications of putative biomarkers of PTSD. Biological markers with sufficient specificity may assist in validating PTSD diagnosis, assessing risk for PTSD, and documenting trauma exposure. Routinely incorporating biological measures into clinical research could also provide invaluable information on the degree of recovery necessary to produce significant and substantial change in underlying pathophysiology of the disorder. Such a change might be a more accurate indicator of recovery and associate with reduced chance of recrudescence. Additionally, treatment outcome studies that incorporate genetic, neuroendocrine, epigenetic, and neurobiological markers may generate predictors of treatment response, recommendations for treatment matching, and new insights that may inform the development of more refined psychological and pharmacologic interventions for the successful treatment of PTSD.

The optimism over the utility of PTSD biomarkers must be tempered by consideration of the potential ethical, social, clinical and legal implications of their applications. The potential implications and applications of biomarkers of disease have been more widely discussed in other areas of medicine where biological markers have been more robustly established, such as Alzheimer's disease (e.g., Gauthier, Leuzy, Racine, & Rosa-Neto, 2013; Illes, Rosen, Greicius, & Racine, 2007; Prvulovic & Hampel, 2011; Slats et al., 2010), but such discussion has been lacking in PTSD research. As the field advances, interdisciplinary perspectives from clinicians, bioethicists and legal scholars should be solicited. The opportunities for biomarkers to improve clinical care and treatment are exciting. They also invite responsible reflection to ensure the greatest good with the least harm.
